# Generalized Trust and Financial Risk-Taking in China – A Contextual and Individual Analysis

**DOI:** 10.3389/fpsyg.2018.01308

**Published:** 2018-07-26

**Authors:** Yi Xu

**Affiliations:** USC-SJTU Institute of Cultural and Creative Industry, Shanghai Jiao Tong University, Shanghai, China

**Keywords:** generalized trust, financial risk-taking, China, inequality, stock market participation

## Abstract

Previous evidence from developed nations has suggested that more trusting individuals are more likely to take financial risks, such as investing in the stock market. Previous studies have found that Chinese citizens have particularly high generalized trust and are more risk-seeking in investment compared with Americans, which makes China an interesting case. The current study examines the relation between generalized trust and stock market participation in China at both a contextual and individual level. Across provinces, a lower level of generalized trust was associated with stock market participation. For example, the stock market participation was four times higher in provinces with the lowest level of perceived fairness than in provinces with the highest level of perceived fairness. The contextual effects of less generalized trust suggest an association between risk-taking behaviors and societal level inequality. At the individual level, trust of strangers was associated with risk preference in highly educated and wealthy people but its effect on risk behaviors was not clear. The findings suggest that trust may affect financial risk-taking behavior at different levels through different pathways, and that cultural differences in understanding of trust also need to be considered.

## Introduction

In the literature, there has been a long discussion about the relationship between trust and risk-taking (Nickel and Vaesen, 2012). For example, when deciding whether to trust someone, there is a risk associated with the other person or entity they rely on to cooperate (Cook and Cooper, 2003). Ben-Ner and Putterman (2001) claim that greater risk aversion can lead to less trust between individuals. Research indicates that trusting behavior is associated with betrayal cost (Bohnet and Zeckhauser, 2004). For instance, people are more reluctant to rely on a human trustee than on a random device offering the same probabilities in a trust game. However, if a situation is inherently risky, people would rely more on trusted relationships than they otherwise would (Kollock, 1994).

More recently, studies have indicated an association between generalized trust and financial risk-taking behavior such as stock market participation (Guiso et al., 2008; Georgarakos and Pasini, 2011). Guiso et al. (2008) examined this issue using a household survey of Dutch citizens. The researchers assessed generalized trust by asking participants questions such as whether most people can be trusted or whether one must be very careful in dealing with people. They also measured risk aversion by asking participants to name the maximum price they would be willing to pay for a ticket to participate in a lottery. The participants were also surveyed on stock market investments. The results showed that risk aversion poorly predicted stock ownership, but also that generalized trust was highly predictive, in that more trusting individuals were more likely to own stocks. Guiso et al. (2008) posited that generalized trust was not simply a proxy for loss aversion. They found different insurance practices between less-trusting people and loss-averse people. People who are more loss-averse are inclined to insure themselves more, while less-trusting people insure themselves less. This observation is also supported by their findings that risk aversion and trust have different predictive power in predicting stock ownership, as described above. To explain the effect of generalized trust, the authors suggested that generalized trust represents a more general trusting relationship between an individual and a financial system, and therefore can lower the cost of financial market participation.

However, most of these results have come from developed nations. For example, Georgarakos and Pasini (2011) found that trust was effective in predicting stockholding in Austria, Spain, and Italy but was not in Sweden, Denmark, or Switzerland, which may relate to differences of education and wealth level. The question remains whether the association between generalized trust and stock market participation holds in other places, particularly in developing nations.

I am specifically interested in examining this question in China for two reasons. First, generalized trust varies substantially across societies internationally, and China is a remarkable outlier. In many European nations, an increase in collectivism is associated with increased close relationships, as well as inhibiting the development of unconditional trust in people’s general benevolence (Gheorghiu et al., 2009). However, China does not follow this pattern of a negative relationship between generalized trust and collectivism (Allik and Realo, 2004; Realo et al., 2008). Among 42 countries analyzed by Allik and Realo (2004), China had the lowest rating of individualism but nearly the highest level of generalized trust, although the authors did not offer an explanation for these findings. The second reason that China is a nation of interest in this issue is because Chinese people are believed to be more risk averse due to the fact that traditional Chinese culture stresses modesty and prudence. Despite the stereotype that Americans are more risk-seeking than Chinese, one risky-decision experiment showed that Chinese people take more risks in investment (Hsee and Weber, 1999). To explain the findings from a cultural perspective, Hsee and Weber proposed the “cushion hypothesis” that Chinese people are more likely than Americans to receive financial help from their close-knit social networks in situations of need, and therefore can take on more financial risk. In conclusion, these studies suggest an atypical pattern of generalized trust and risk attitudes in China, raising the question of whether the association of generalized trust and stock market participation is different in China.

In the current study, I examined the association between generalized trust and stock market participation at both the contextual and individual levels. Previous studies indicate that generalized trust can influence people not only at the individual level but also at the contextual level. For example, state-level generalized trust can effectively predict individuals’ self-rated health (Kawachi et al., 1999). At the contextual level, trust may influence people’s behavior through social norms and interactions within the community. Evers and Gesthuizen (2011) also found that the effect of trust on prosocial behaviors varies at the individual and country levels with different mechanisms. Presumably, generalized trust influences stock market participation at different levels. Regional economic differences in China have a significant effect on generalized trust and independence (Takemura et al., 2016). Thus, I propose that at the contextual level generalized trust influences stock market participation in China.

In addition, Georgarakos and Pasini (2011) found that the effect of trust in predicting stock market participation was weaker in countries with higher levels of trust. The authors also observed that generalized trust had no effect on less wealthy households in low participation countries. Likewise, Guiso and Jappelli (2004) documented that a significant fraction of Italian households limited their stock market participation due to financial ignorance, which is associated with a lack of education and wealth. It is likely that in China, a developing country with a low market participation rate (8.96% in 2010), many people could fit the profile of less wealthy households with low awareness of financial assets. Therefore, considering China’s high level of generalized trust and low financial awareness, I suggest that the positive effect of generalized trust on stock market participation at individual level may not be upheld in China.

In study 1, I used data from two national surveys to explore how contextual generalized trust can be identified for individual stock market participation. In study 2, an online survey was used to examine the association between individual level generalized trust and stock market participation. Together, these studies can serve as the first evidence that examines the relationship between generalized trust and stock market participation in China and can help understand generalized trust and risk attitudes under different cultural context.

## Study 1

### Materials and Methods

Study 1 examined the association between contextual-level generalized trust and stock market participation. Two national surveys were used in the study: the Chinese Household Finance Survey [CHFS], 2010 and Chinese General Social Survey [CGSS], 2011. The aim of the CHFS is to examine household finance conditions, and the aim of the CGSS is to monitor changes in social structure and quality of life. Both surveys used stratified sampling covering urban and rural China, and interviews were conducted by trained staff. The CHFS was administrated through 80 counties in 25 provinces in China in 2011. The sample size was 8500 households, which were selected to match overall Chinese economic, geographic and urbanization distributions. The distribution of per capita GDP of the sample counties was similar to the distribution in China overall. The ratio of counties selected in Eastern, Central, and Western China was 32:27:21, closely matching the overall geographic distribution of counties in China. In 2010, the CGSS was administered in 31 provinces, including 100 counties and 5 metropolises. The sample size was 12,000 households. Twenty-five provinces covered in both surveys were selected in the current analysis, including four municipalities (Beijing, Shanghai, Tianjin, and Chongqing), eight more-developed eastern provinces (Jiangsu, Zhejiang, Guangdong, Anhui, Heilongjiang, Jilin, Liaoning, Shandong), six provinces in central areas (Hebei, Henan, Hubei, Hunan, Jiangxi, Shanxi), and seven provinces in relatively underdeveloped western rural areas (Sichuan, Shaanxi, Guangxi, Gansu, Qinghai, Yunan, Guizhou).

#### Generalized Trust

Barber (1983) claims that trust implies positive expectation of the trustee’s honesty, trustfulness, fairness, and willingness to help others. Likewise, generalized trust is generally understood to refer to concepts such as trustfulness (whether most people are trustworthy), caution (whether most people would take advantage of you), fairness (whether others are fair to you), and helpfulness (people are mostly trying to be helpful). Previous measures such as the American General Social Survey and the European Social Survey have evaluated these concepts. Three items from the CGSS were used to measure generalized trust and a 5-point Likert scale was used for the scoring. Respondents were presented with the statements, “Generally, do you agree that most people in society are trustworthy?” and “Generally, do you agree that if you are not careful, other people will take advantage of you?” (1 = very much disagree, 5 = very much agree), as well as “Generally, do you think that current society is fair?” (1 = very unfair, 5 = very fair). Although a previous study comparing European countries and utilizing a similar set of questions revealed a reliable one-factor solution (for 31 countries, alphas ≥ 0.60) (Gheorghiu et al., 2009), I conducted a factor analysis with individual-level data and found the reliability of the three items was not satisfactory (alpha = 0.47). Therefore, in the following analysis, I examined the three generalized trust items separately across provinces: trust (*M* = 3.52, *SD* = 0.17), caution (reverse coded, *M* = 2.97, *SD* = 0.18), and fairness (*M* = 2.98, *SD* = 0.21). The individual-level data were adjusted with weights provided by the CGSS. The variables were coded so that higher scores indicate a higher level of trust. Provinces were grouped into three levels (high, medium, or low) of each generalized trust item based on a cut point defined by onw standard deviation on either side of the overall mean (See Supplementary Table 1 for each province’s generalized trust level).

#### Risk Preference and Stock Market Participation

Evidence suggests that a simple risk-attitude question can produce reliable results (Dohmen et al., 2011). Risk preference was assessed by the item in the CHFS, “Assume you have some assets to invest. Which type of project would you invest in?” Respondents indicated their choice by choosing 1 (“high risk, high return”), 2 (“slightly above-average risk, slightly above-average return”), 3 (“average risk, average return”), 4 (“slightly below-average risk, slightly below-average return”), or 5 (“unwilling to take any risk”)^1^. A similar question was used in the Survey of Consumer Finances (SCF) in the United States and prior literature has documented that this risk-attitude question predicts actual household allocation to risky assets (e.g., Shum and Faig, 2006; Malmendier and Nagel, 2011).

Stock market participation is captured by the question of whether the household has one or more brokerage accounts for stock trading. Based on the CHFS, nationwide, 8.96% of households in China have brokerage accounts. Across provinces, the mean risk attitude in investment is 2.17 (reverse coded, a higher score indicates more risk-seeking, *SD* = 0.20), and the mean percentage of respondents holding brokerage accounts for stock trading is 7.62% (*SD* = 8.34%).

#### Demographic Variables

Demographic variables from the CHFS – age, marital status, gender, education, and household income were included in the analysis. Individual demographic information of the head of household, such as gender, age, and education was used. Head of household was defined in the CHFS as the person with the most accurate knowledge about household finances. Annual household income was collected and ranked as a measure of relative income level. A broader body of research work has indicated that these factors are positively correlated with risk preference: male (vs. female), married (vs. unmarried), younger (vs. older), higher education (vs. lower education), and higher income (vs. lower income) (e.g., Friend and Blume, 1975; Grable, 2000; Croson and Gneezy, 2009; Dohmen et al., 2011).

### Results

**FIGURE 1:**
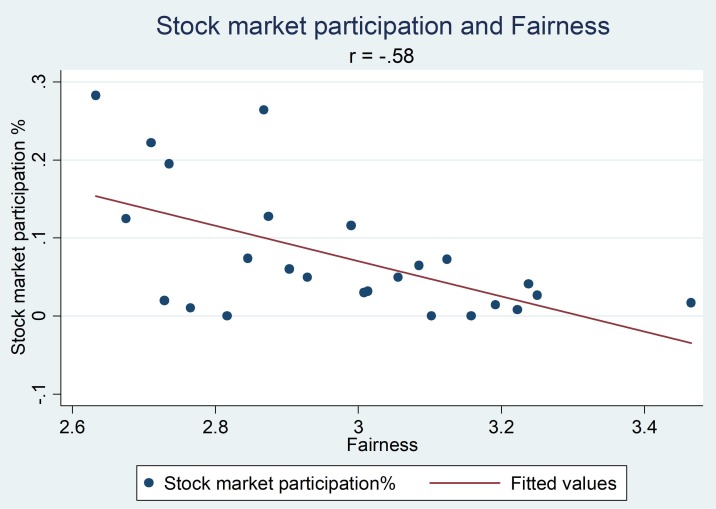
Scatterplot of levels of generalized trust indicator – fairness and percentage of stock market participation at the provincial level.

**Table 1** shows the descriptive analysis of each variable and percentage of subjects participating in the stock market. Consistent with previous findings, stock market participation was associated with more risk-taking attitude, higher income and higher education level (Guiso et al., 2008; Dohmen et al., 2011). At the provincial level, three indicators of generalized trust were each negatively associated with stock market participation: fairness (*r* = −0.58, *p* = 0.002) (**Figure 1**), caution (*r* = −0.49, *p* = 0.014), and, though less significant, trust (*r* = −0.32, *p* = 0.12). Among the three indicators, trust and fairness showed a significant correlation with one another (*r* = 0.75, *p* < 0.001), but caution was not associated with the other two indicators (*r*_trust_ = 0.02, *p* = 0.92, *r*_fairness_ = 0.29, *p* = 0.15).

**Table 1:** Descriptive analysis of variables and association with stock market participation.

	% (*N* = 8241)	% Participate stock market
**Head of household gender**		
Male	53.9	8.0
Female	46.1	10.0
Married family	85.5	9.0
**Age**		
35 years and younger	19.0	13.7
35–44	25.2	11.9
45–54	22.8	7.8
55–64	19.3	5.7
66–74	9.7	4.1
75 years and older	4	2.1
**Education**^a^		
No high school diploma	64.4	2.9
High school diploma	13.7	11.2
Some college/occupation school	13.9	21.5
College and above	8.0	32.7
**Income rank (RMB)**		
Bottom 20%	<8.3 K	2.5
Lower middle 20%	8.3–20.5 K	2.4
Upper middle 40%	20.5–63.6 K	7.4
Top 20%	>63.6 K	25.1
**Risk preference**		
Highly risk seeking	6.2	17.6
Risk seeking	7.3	22.7
Risk neutral	25.9	11.5
Risk averse	16.9	9.2
Highly risk averse	43.7	3.8
**Residence in province characterized by**		
Low trust	21.5	10.9
Medium trust	70.9	9.0
High trust	7.7	3.0
Low caution	9.0	4.0
Medium caution	91.0	9.2
High caution	1.2	28.3
Low fairness	22.8	16.2
Medium fairness	63.1	7.9
High fairness	14.1	2.2

*^a^Education data missing in 77 individuals.*

First, logit regression was used to examine stock market participation with individual level variables (**Table 2**), and then province-level generalized trust indicators were added (**Table 3**). The strongest associations with stock market participation were education and income. People with a college degree or higher were seven times more likely to trade stock compared with people without a high school education [OR = 7.47, 95% (CI) = 5.72, 9.76]. Similarly, people who were within the top 20% income group of CHFS were five times more likely to trade stock compared with people whose incomes were ranked in the bottom 20% [OR = 5.24, 95% (CI) = 3.70, 7.43]. Risk preference also significantly predicted stock market participation in that risk-seeking people were three times more likely to trade stock than people who were highly risk averse [OR = 3.59, 95% (CI) = 2.66, 4.85]. At the contextual level, a lower level of generalized trust was associated with higher levels of stock market participation. People living in places with the lowest level of fairness were four times more likely to trade stocks compared with people living in areas with highest level of fairness [OR = 4.29, 95% (CI) = 2.77, 6.64]. People in provinces with higher levels of caution and lower levels of trust were also more likely to participate in the stock market [for highest caution vs. lowest: OR = 2.48, 95% (CI) = 1.31, 4.66; for lowest trust vs. highest: OR = 2.08, 95% (CI) = 1.25, 3.47]. An additional analysis using quartile cutpoints of generalized trust provided the same pattern with a stronger gradient^2^.

**Table 2:** Logistic regression analysis of stock market participation with individual level variables.

		OR	SE	*z*	Significnce	95% Confidence Interval
Head of Household	Male	1.00				
	Female	1.57	0.14	5.11	^∗∗∗^	(1.31,1.86)
Marital	Married	1.00				
	Unmarried	1.03	0.13	0.19	0.84	(0.79,1.32)
Age	<35 years	1.00				
	35–44	1.68	0.20	4.44	^∗∗∗^	(1.34,2.12)
	45–54	1.45	0.20	2.75	^∗∗^	(1.11,1.90)
	55–64	1.60	0.25	3.03	^∗∗^	(1.18,2.17)
	66–74	1.09	0.24	0.42	0.67	(0.72,1.67)
	≥75 years	0.54	0.22	–1.47	0.14	(0.24,1.22)
Education	No high school diploma	1.00				
	High school diploma	3.09	0.41	8.51	^∗∗∗^	(2.38,3.99)
	Some college/occupation school	5.53	0.66	14.40	^∗∗∗^	(4.38,6.98)
	College and above	7.47	1.02	14.78	^∗∗∗^	(5.72,9.76)
Income	Bottom 20%	1.00				
	Lower middle 20%	0.90	0.21	-0.47	0.64	(0.57,1.41)
	Upper middle 40%	1.98	0.35	3.85	^∗∗∗^	(1.39,2.80)
	Top 20%	5.24	0.93	9.30	^∗∗∗^	(3.70,7.43)
Risk preference	Highly risk averse	1.00				
	Risk averse	1.69	0.23	3.79	^∗∗∗^	(1.29,2.22)
	Risk neutral	1.86	0.23	4.99	^∗∗∗^	(1.46,2.38)
	Risk seeking	3.59	0.55	8.36	^∗∗∗^	(2.66,4.85)
	Highly risk seeking	3.46	0.57	7.48	^∗∗∗^	(2.50,4.79)
*N*						8158
*Df*						17
LRχ^2^						1139.34
Pseudo *R^2^*						0.23

*^∗^p < 0.05, ^∗∗^ p < 0.01, ^∗∗∗^ p < 0.001.*

**Table 3:** Logistic regression analysis of stock market participation with province level generalized trust after all individual level variables were controlled.

		OR	SE	*z*	Significance	95% Confidence Interval
Trust	Low	2.08	0.54	2.82	^∗^	(1.25,3.47)
	Medium	1.77	0.44	2.29	^∗∗^	(1.09,2.90)
	High	1.00				
Caution	Low	2.48	0.80	2.81	^∗∗^	(1.31,4.66)
	Medium	1.85	0.37	3.04	^∗∗^	(1.24,2.74)
	High	1.00				
Fairness	Low	4.29	0.96	6.53	^∗∗∗^	(2.77,6.64)
	Medium	2.39	0.52	4.00	^∗∗∗^	(1.56,3.66)
	High	1.00				

*^∗^p < 0.05, ^∗∗^p < 0.01, ^∗∗∗^ p < 0.001.*

Next, the effect of the economic development of each province was considered. Provincial level GDP per capita in 2010 was added into the regression, which positively predicted stock market participation [OR = 1.05, 95% (CI) = 1.04, 1.07]. After GDP per capita was controlled for, the effect of generalized trust remained though attenuated. Conditions such as lower fairness still significantly predicted stock market participation, whereas the odds ratio for the least fair province compared with the most fair province was 2.91 [95% (CI) = 1.83, 4.65]. Last, the Gini coefficient representing the income distribution of each province in 2010 was used as a measurement of income inequality. After the individual level of variables were controlled for, the Gini coefficient significantly predicted stock market participation (OR = 37.54, *SD* = 35.17, *z* = 3.87, *p* < 0.001). However, with fairness included in the regression, the Gini coefficient was no longer significantly associated with stock market participation (OR = 0.46. *SD* = 0.56, *z* = −0.64, *p* = 0.52). It seems that fairness captures the contextual effect of income inequality.

This result differs from Guiso et al. (2008), who concluded that trust had a positive effect on stock market participation. It seems that the contextual effects of less generalized trust are associated with more financial risk-taking.

## Study 2

Study 2 examined the association between individual level generalized trust, risk preference, and stock market participation. A measurement of trust in strangers was added to examine its effect on stock market participation.

An online survey was conducted with 422 individuals via a Chinese survey platform^3^. The platform has more than two million users nationwide. The 422 individuals were from 27 provinces including provinces in the east (73.7%), such as Shanghai (10.0%), Jiangsu (10.4%), Guangdong (13.0%), and Beijing (11.8%) as well as other provinces in the central area (10.7%) and western area (15.9%).

### Materials and Methods

Generalized trust was assessed by the same questions as used in Study 1 with a 7-point Likert scale: trust (*M* = 4.77, *SD* = 1.10), caution (reverse coded, *M* = 2.74, *SD* = 1.26), and fairness (*M* = 4.49, *SD* = 1.28). Additionally, a question that measured trust of strangers was included (“Generally, do you agree that most strangers can be trusted?”). It should be noted that Chinese respondents may interpret “most people” (the terms used in the measurement of generalized trust) as people they know (Tang, 2005). To a certain extent, the trust question may assess their trust of familiar people instead of generalized trust that extends to strangers. Consistent with Tang (2004), this was supported by the evidence of significantly lower levels of trust of strangers in this study (*M* = 3.81, *SD* = 1.26, *t* = −16.5, *df* = 421, *p* < 0.001).

Risk preference was measured by investment scenario questions (Hsee and Weber, 1999). Respondents were asked to choose from two investment options – buy a stock whose return rate was equally likely to be 0 or 8%, or put the money in a savings account in which the return rate was fixed. The saving return rates were 2, 4, and 6% in three scenarios. The risk preference was calculated based on their choices in the three scenarios from 1 (most risk-averse, if a participant chose the saving option in all three questions) to 4 (most risk-seeking, if a participant chose the risk options in all three questions). Participants whose choice pattern was inconsistent (e.g., choosing 2% saving over stock but choosing stock over 6% saving) were assigned a missing value and excluded in the further analysis (29 participants).

Stock market participation was captured by the same question of whether the household has one or more brokerage accounts for stock trading. Demographic variables – age, marital status, gender, education, annual household income were included in the analysis.

### Results

A descriptive analysis showed that the sample was composed of middle aged people (*M* = 34.2, *SD* = 8.31, 50.5% between 25 and 34 years old), with high education (73.5% with college and above), relatively high annual household income (*M* = 195 k, *SD* = 83.1), and much higher rate of stock market participation (44.1% vs. 8.96% nation-wide).

A logistical regression was conducted to examine the association between stock market participation, risk preference, and trust indicators stock market participation was only associated with trust of strangers (*r* = 0.11, *p* = 0.025) but not with other trust variables. After all demographic variables were controlled for, none of the trust indicators significantly predicted stock market participation. Rather, such participation was mainly predicted by risk preference (**Table 4**). In fact, the most risk-seeking people were nineteen times more likely to trade stock than people who were the most risk averse (OR = 19.72, 95% [CI] = 4.63, 84.14).

**Table 4:** Logistic regression analysis of stock market participation with risk preference and demographic variables.

		%	OR	SE	*z*	Significance	95% CI
Gender	Male	50.5	1.00				
	Female	49.5	1.02	0.25	0.10	0.92	(0.63,1.65)
Marital	Married	83.2	1.00				
	Unmarried	16.8	0.37	0.13	-2.73	^∗∗∗^	(0.18,0.75)
Age	<25 years	11.4	1.00				
	25–34	50.5	1.72	0.76	1.23	0.22	(0.73,4.08)
	35–44	26.5	2.39	1.16	1.80	0.07	(0.93,6.18)
	≥44 years	11.6	2.46	1.39	1.58	0.11	(0.81,7.48)
Education	High school and below	7.3	0.13	0.09	–2.89	^∗^	(0.03,0.52)
	Some college/occupation school	19.2	0.50	0.16	–2.16	^∗∗^	(0.26,0.94)
	College and above	73.5	1.00				
Income (RMB)	≤100 k	21.1	1.00				
	100–150 k	21.1	3.12	1.25	2.85	^∗∗^	(1.43,6.84)
	150–250 k	37.9	1.97	0.74	1.80	0.07	(0.94,4.11)
	>250 k	19.9	1.91	0.81	1.52	0.13	(0.83,4.37)
Risk preference	Most risk averse	16.4	1.00				
	Risk averse	37.9	5.17	2.66	3.18	^∗∗^	(1.88,14.19)
	Risk seeking	41.7	18.52	9.54	5.66	^∗∗∗^	(6.74,50.84)
	Most risk seeking	4.0	19.72	14.6	4.03	^∗∗∗^	(4.63,84.14)
*N*							422
*df*							13
LRχ^2^							130.23
Pseudo *R^2^*							0.23

*^∗^p < 0.05, ^∗∗^p < 0.01, ^∗∗∗^p < 0.001.*

Finally, I looked into the relationship between risk preferences and trust indictors. Risk preference was positively correlated with trust of most people (*r* = 0.11, *p* = 0.027) and trust of strangers (*r* = 0.16, *p* < 0.001). Ordered logistic regression was used to analyze the association between risk preference and trust indicators. After all demographic variables were controlled for, only trust of strangers significantly predicted risk preference [OR = 1.21, *p* = 0.012, 95% (CI) = 1.04, 1.40]. Specifically, one-unit increase in trust of strangers was associated with 21% increased odds of risk-seeking.

In sum, these findings provide evidence that at an individual level, stock market participant can be predicted by risk preference but not by generalized trust, although trusting strangers can increase risk tolerance.

## General Discussion

The current study examined the effect of generalized trust on stock market participation in China. At the individual level, people who are more risk-seeking in investment, wealthier and with higher education tend to participate in the stock market more often. At the contextual level, people living in areas with lower levels of generalized trust are more likely to invest in the stock market compared to people living in areas with higher levels of generalized trust. It is possible that generalized trust may affect individual financial risk-taking behavior through different pathways, depending on the level at which it is measured.

### Contextual Level: Societal Level Inequality and Risk-Taking Behavior

At the contextual level, among the indicators of generalized trust, the effect of fairness was obvious. Researchers argue that fairness at the societal level is important in generating social trust (e.g., Yamagishi, 1998; Stolle, 2002), while lower levels of fairness indicates an issue of inequality. Societal level inequality is linked to risk-taking behavior. Wilkinson and Pickett (2006, 2007, 2009) have reviewed the literature indicating that economic inequality has been associated with many negative outcomes including poor physical and mental health, poor educational outcomes, and higher levels of violence and criminal activity. Many of these negative outcomes have been associated with individual risk-taking behaviors, such as violence (Morenoff et al., 2001), drug and substance abuse (Room, 2005), and other crimes (Daly et al., 2001).

The association between inequality and risk-taking behavior can be explained from an evolutionary perspective. People need resources to survive. When people find the distribution of outcomes is unfair and that they are in a disadvantaged position, or when they find disparity between their present state and their desired state, they are triggered to taking high variance options in decision making (Stephens, 1981; Stephens and Krebs, 1986; Mishra and Lalumière, 2010). The level of inequality is not only decided by economic income but also by subjective experience of inequality. Individuals with identical incomes can perceive or experience inequality differently according to their living contexts (Luttmer, 2005). In the current study, perceived inequality is indicated by a lower level of generalized trust at the provincial level. This lack of trust is then associated with greater financial risk-taking behavior. The finding of Study 1 is consistent with evidence that at the contextual level, a feeling of less fairness makes people participate more in the stock market, as in Payne et al. (2017).

At the individual level, it seems that the effect of trust on stock market participation can be moderated by individual level variables such as wealth and education. In the previous findings, Guiso et al. (2008) also indicated that the effect of generalized trust on stock market participation was insignificant for highly educated investors. This is consistent with the results of Study 2. Due to the sample limitation, conclusions cannot be drawn as to whether individual level generalized trust in China positively impacts stock market participation or not. However, study 2 suggests that the understanding of generalized trust also matters.

### Understanding Generalized Trust in a Cultural Context

Cultural context needs to be taken into consideration in the understanding of generalized trust (Gheorghiu et al., 2009), though very limited research is available. For example, empirical evidence suggests that Japanese people have a different understanding of trust compared with Americans (Yamagishi and Yamagishi, 1994). Researchers have proposed that generalized trust in China may be inflated by the authoritarian political system (Bjørnskov, 2006; Steinhardt, 2012). While other Chinese scholars have argued that as Chinese people are tightly connected with family and friends, the feeling of trust of outsiders appears so alien that survey respondents may not include strangers in their interpretation of “most people” (Tang, 2004, 2005). The results of Study 2 support this view. Thus, a question that specifies trust of strangers rather than trust of most people can more accurately assess generalized trust in China. Additionally, previous research has proposed that caution does not necessarily lead to distrust. In particular, Japanese and American citizens can have quite different understandings of caution, which may imply cultural difference in the interpretation of this concept (Miller and Mitamura, 2003). This is consistent with the result of Study 1 that the three indicators – trust, caution, and fairness may reflect slightly different elements of trust. For example, caution was not strongly correlated with the other two indicators in Study 1.

Earlier studies have tried to understand the relationship between risk attitude and trust (e.g., Eckel and Wilson, 2004). However, very few studies have examined this question in Eastern countries. One field study examined the relationship between trusting behavior and generalized trust with rural farmers in China (Tu and Bulte, 2010). They found that generalized trust positively predicted actual risk-taking behavior in the field. The current result also indicated that at an individual level, risk attitude in investment was associated with trust of strangers among Chinese citizens. Future research could try to understand the relationship between risk and trust, taking cultural context into account (Christopoulos and Tobler, 2016).

### Limitations

There are several limitations worth acknowledging in the present two studies. First, Study 1 used datasets from two national surveys. Both surveys used stratified sampling, and the combined datasets covered 25 provinces, which is quite representative of the general population in China. However, the datasets do not allow analysis of generalized trust at an individual level. Research examining the effect of social capital emphasizes the importance of contextual level analysis because social relationships measured at the individual level may not capture group-level processes, such as the collective of features of society. For example, Kawachi et al. (1999) used U.S. data aggregated at the state level and reported strong correlations between generalized trust and morality rates. The results of Study 1 should be interpreted as contextual effect of generalized trust. Additionally, although the aggregated data of generalized trust was adjusted with weights provided by CGSS, the weights may not be best representative at the province level^4^.

Second, the results of Study 2 were limited by sample bias. Though the samples were from 27 provinces of China, the majority of participants was from the most developed areas and had high levels of education and high household incomes. The findings support the view that the effect of generalized trust on stock market trading can be moderated by individual differences such as education level.

Third, the interpretation of the result is limited to associations and not causation. Future studies should investigate the potential causal relationship between trust and risk-taking. Additionally, the mechanism between trust and risk-taking stills needs to be investigated in the context of culture.

## Conclusion

In conclusion, stock market participation in China is associated with risk preference and generalized trust. At the contextual level, a lower level of generalized trust was associated with stock market participation. It seems that the lower level of fairness perceived as inequality across provinces encourages people to be more involved in financial risk-taking behavior. Trust of strangers was associated with risk preference in highly educated and wealthier people. To understand the relationship between generalized trust and financial risk-taking, cultural differences need to be considered.

## Ethics Statement

This study (Study 2) was carried out in accordance with the recommendations of Survey and Behavioural Research Ethics Committee of ICCI, Shanghai Jiao Tong University; with written informed consent from all subjects. All subjects gave written informed consent in accordance with the Declaration of Helsinki. The protocol was approved by the Survey and Behavioural Research Ethics Committee of ICCI, Shanghai Jiao Tong University. The data reported in this manuscript Study 1 were obtained from publicly available data, the Chinese Household Finance Survey (CHFS) (http://www.chfsdata.org/) and the Chinese General Social Survey (CGSS) (http://www.chinagss.org/).

## Author Contributions

The author confirms being the sole contributor of this work and approved it for publication.

## Conflict of Interest Statement

The author declares that the research was conducted in the absence of any commercial or financial relationships that could be construed as a potential conflict of interest.

## Footnotes

1 To enable comparison of the pattern of risk preference to previous research, the responses were categorized as “risk averse” (options 4 and 5), “risk neutral” (option 3), and “risk seeking” (options 1 and 2). Most respondents, 57.27% were risk averse, while 28.37% were risk neutral and 14.36% were risk seeking. This pattern was similar to what Weber and Hsee (1998) found in Chinese participants, which was 67, 21, and 12%, respectively.

2 In a regression analysis that the three indicators of generalized trust were kept as continuous variables, same results were obtained that lower levels of generalized trust significantly predicted higher levels of stock market participation.

3 www.sojump.com

4 The CHFS and CGSS used similar stratified sampling methods. The demographic variables (gender, age, education, and household income) of 25 provinces covered in both surveys were compared and showed no significant differences.
