# Supplementary material for: Generalized Trust and Financial Risk-Taking in China – A Contextual and Individual Analysis
Source: Front Psychol. 2018 Jul 26;9:1308. doi: 10.3389/fpsyg.2018.01308 (PMC6070696; doi:10.3389/fpsyg.2018.01308)
Supplement: Supplementary file 1 [file Table_1.docx]

**Appendix**

Table 1. Generalized trust of each province.

| Province | N | Trust | Trust level | Caution^a^ | Caution level | Fairness | Fairness Level |
| --- | --- | --- | --- | --- | --- | --- | --- |
| Anhui | 409 | 3.69 | Medium | 2.00 | Medium | 3.12 | Medium |
| Beijing | 518 | 3.49 | Medium | 1.80 | Medium | 2.87 | Medium |
| Chongqing | 298 | 3.70 | High | 1.85 | Medium | 3.47 | High |
| Gansu | 200 | 4.05 | High | 1.96 | Medium | 3.25 | High |
| Guangdong | 547 | 3.32 | Low | 1.98 | Medium | 2.74 | Low |
| Guangxi Zhuang | 393 | 3.52 | Medium | 2.10 | Medium | 3.10 | Medium |
| Guizhou | 302 | 3.34 | Low | 1.81 | Medium | 2.82 | Medium |
| Hebei | 291 | 3.56 | Medium | 2.08 | Medium | 3.01 | Medium |
| Heilongjiang | 609 | 3.55 | Medium | 2.11 | Medium | 3.09 | Medium |
| Henan | 575 | 3.39 | Medium | 2.04 | Medium | 3.06 | Medium |
| Hubei | 615 | 3.65 | Medium | 2.03 | Medium | 3.01 | Medium |
| Hunan | 485 | 3.32 | Low | 2.01 | Medium | 2.85 | Medium |
| Jiangsu | 504 | 3.45 | Medium | 2.01 | Medium | 2.73 | Low |
| Jiangxi | 469 | 3.47 | Medium | 2.20 | High | 2.93 | Medium |
| Jilin | 487 | 3.66 | Medium | 1.97 | Medium | 3.19 | High |
| Liaoning | 398 | 3.33 | Low | 2.20 | High | 2.90 | Medium |
| Qinghai | 100 | 3.46 | Medium | 1.33 | Low | 2.63 | Low |
| Shaanxi | 405 | 3.59 | Medium | 1.87 | Medium | 3.16 | Medium |
| Shandong | 574 | 3.62 | Medium | 2.04 | Medium | 2.99 | Medium |
| Shanghai | 522 | 3.36 | Medium | 2.01 | Medium | 2.71 | Low |
| Shanxi | 293 | 3.33 | Low | 1.92 | Medium | 2.77 | Medium |
| Sichuan | 597 | 3.71 | High | 1.91 | Medium | 3.24 | High |
| Tianjin | 396 | 3.42 | Medium | 1.80 | Medium | 2.68 | Low |
| Yunnan | 385 | 3.68 | Medium | 2.15 | High | 3.22 | High |
| Zhejiang | 569 | 3.36 | Medium | 2.07 | Medium | 2.87 | Medium |
| Total | 10941 |  |  |  |  |  |  |

^a^ Caution was reverse coded.
